# Supplementary material for: Chemical Composition of Turmeric (Curcuma longa L.) Ethanol Extract and Its Antimicrobial Activities and Free Radical Scavenging Capacities
Source: Foods. 2024 May 16;13(10):1550. doi: 10.3390/foods13101550 (PMC11121704; doi:10.3390/foods13101550)
Supplement: Supplementary file 1 [file foods-13-01550-s001.zip › foods-2998792-SI.pdf]

Supplementary Figure S1

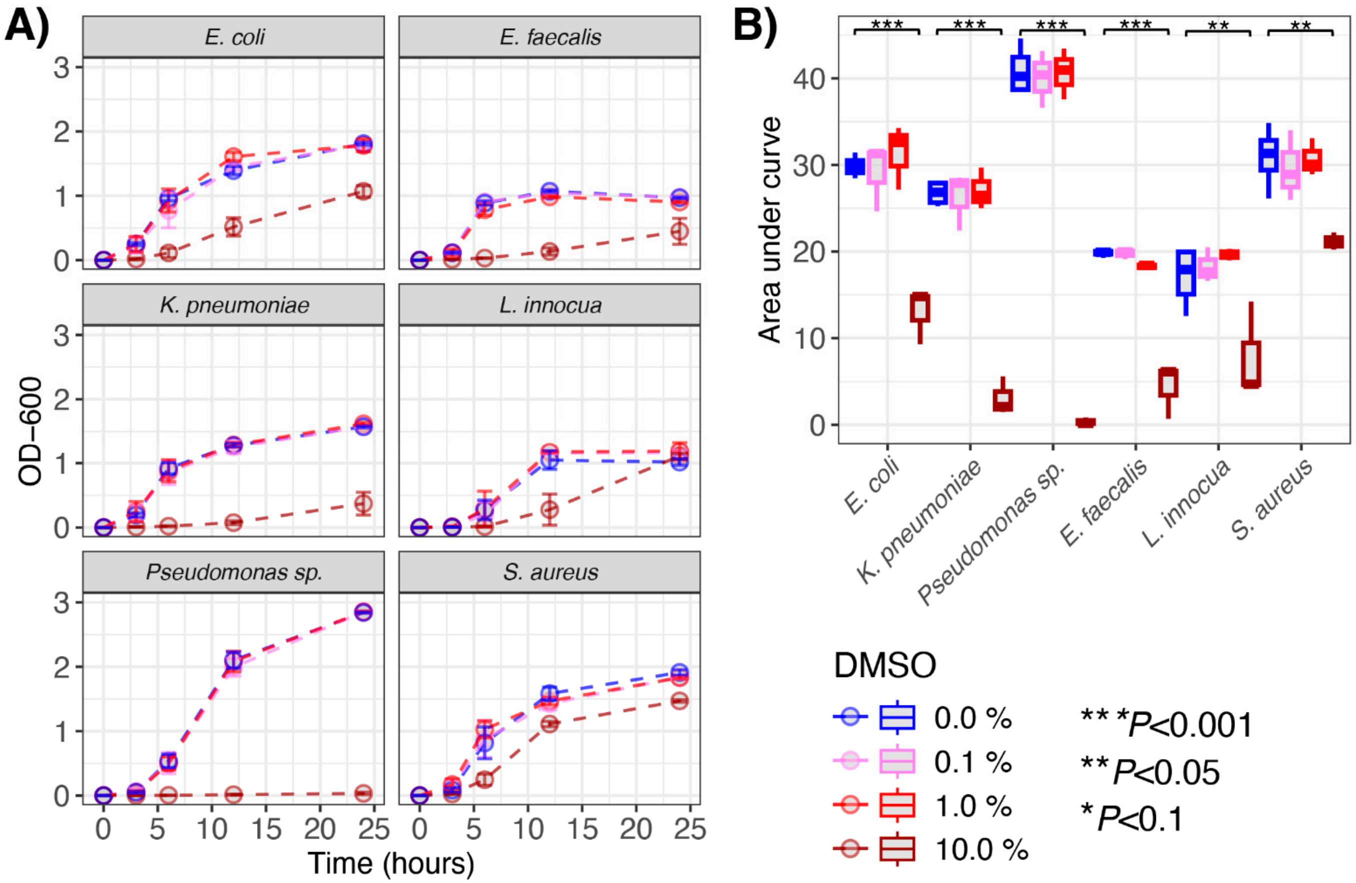

Supplementary Figure S1. Effects of DMSO on bacterial growth. Panel A shows the growth curves and Panel B shows area under the curve with '* and **' for inhibition relative to the controls (0%).
